# The Role of Language and Cultural Adaptation in Access to Mental Health Services among Turkish Migrants: Evidence from NPZ Munich

**DOI:** 10.1192/j.eurpsy.2026.11456

**Published:** 2026-07-31

**Authors:** E. D. Cindik-Herbrueggen, A. S. Kucukali

**Affiliations:** 1 Neuro-Psychiatrisches Zentrum Riem, Munich, Germany; 2 Psychology, Istanbul Gelisim University, Istanbul, Türkiye

## Abstract

**Introduction:**

Turkish-origin migrants form a substantial community in Germany yet face persistent barriers to mental health care. Language proficiency and cultural adaptation are pivotal for engagement and continuity, and culturally sensitive services are recommended (Schouler-Ocak et al. *Bundesgesundheitsblatt* 2015;58 527–532; Mewes et al. *Compr Psychiatry* 2015;62 42–50; Matthew et al. *Int J Res Publ Rev* 2025;6 2984–2997). Empirical evidence on how these factors shape clinic choice remains limited. We examined why Turkish clients select NPZ Munich, a bilingual psychiatric clinic, and how linguistic/cultural factors relate to adherence.

**Objectives:**

To identify linguistic and cultural determinants of clinic preference and treatment adherence among Turkish migrants in Munich.

**Methods:**

We conducted a descriptive, cross-sectional mixed-methods study (n=250) of Turkish patients who attended NPZ Munich (2020–2025). Quantitative data from standardized intake forms included demographics, duration of treatment, and pharmacological history; we calculated frequencies, percentages, and mean treatment-seeking delay (months). Qualitative data from open-ended responses underwent thematic analysis to identify recurrent patterns. All data were de-identified; procedures complied with institutional privacy and GDPR requirements.

**Results:**

Quantitatively, 62% had been in treatment >2 years; 48% reported initiating pharmacotherapy at NPZ Munich. The average delay from symptom onset to first presentation was 8.5 months. Qualitative analysis yielded four themes shaping clinic preference: (1) language/mother-tongue access (“I chose it because Turkish is spoken”); (2) cultural familiarity/belonging (“they understand my culture better”); (3) recommendations and trust (family/friend referrals); and (4) availability and accessibility (prolonged search before suitable care). Findings align with prior work underscoring the value of culturally attuned services.

**Conclusions:**

Language access and cultural proximity appear central to service utilization among Turkish migrants, supporting models that integrate bilingual staff, interpreter support, and culture-informed care pathways. Scaling such services may enhance trust, continuity, and satisfaction while reducing treatment gaps. Policy implications include funding professional interpreters, training clinicians in cultural competence, and adapting this model to other regions with large migrant populations.

**Disclosure of Interest:**

None Declared

